# Thymol Ameliorates *Aspergillus fumigatus* Keratitis by Downregulating the TLR4/ MyD88/ NF-kB/ IL-1&beta; Signal Expression and Reducing Necroptosis and Pyroptosis

**DOI:** 10.4014/jmb.2207.07017

**Published:** 2022-12-01

**Authors:** Limei Wang, Haijing Yan, Xiaomeng Chen, Lin Han, Guibo Liu, Hua Yang, Danli Lu, Wenting Liu, Chengye Che

**Affiliations:** 1 Department of Ophthalmology, the Affiliated Hospital of Qingdao University, Qingdao, Shandong Province 266003, P.R. China; 2 Department of Ophthalmology, Qingdao Women and Children’s Hospital, Qingdao, Shandong Province 266034, P.R. China; 3 Gout Laboratory, the Affiliated Hospital of Qingdao University, Qingdao, Shandong Province 266003, P.R. China

**Keywords:** Thymol, *A. fumigatus* keratitis, necroptosis, pyroptosis, inflammation

## Abstract

Fungal keratitis is a refractory kind of keratopathy. We attempted to investigate the anti-inflammatory role of thymol on *Aspergillus fumigatus* (*A. fumigatus*) keratitis. Wound healing and fluorescein staining of the cornea were applied to verify thymol’s safety. Mice models of *A. fumigatus* keratitis underwent subconjunctival injection of thymol. The anti-inflammatory roles of thymol were verified by hematoxylin-eosin (HE) staining, slit lamp observation, quantitative real-time polymerase chain reaction (qRT-PCR), and Western blotting. In contrast with the DMSO group, more transparent corneas and less inflammatory cells infiltration were detected in mice treated with 50 μg/ml thymol. Thymol downregulated the synthesis of TLR4, MyD88, NF-kB, IL-1β, NLRP3, caspase 1, caspase 8, GSDMD, RIPK3 and MLKL. In summary, we proved that thymol played a protective part in *A. fumigatus* keratitis by cutting down inflammatory cells aggregation, downregulating the TLR4/ MyD88/ NF-kB/ IL-1β signal expression and reducing necroptosis and pyroptosis.

## Introduction

Fungal keratitis (FK) is an acute vision-threatening keratitis that usually occurs from filamentous fungi or yeasts infection after corneal abrasions, typically associated with plant-based materials and contact lens [1, 2]. At present, first-line therapy of FK remains antifungal drugs (natamycin, voriconazole), but the poor effect will still progress to perforation and blindness [1]. Therefore, new preparations are urgently needed to improve the status of refractory fungal keratitis.

Pathogenic fungi enter the cornea through corneal epithelial defect [3]. TLRs, as an pattern recognition receptors (PRRs), take effect on the recognition of *Aspergillus fumigatus* invasion by corneal stromal cells. TLR4 mainly conducts signal transduction through MyD88-dependent and independent pathways. In the MyD88-dependent pathway, after TLR4 binds to its corresponding ligand, it activates the MyD88 protein and promotes the translocation of NF-kB into the nucleus, thereby activating the downstream related gene transcription process, and activating macrophages, monocytes, etc. to synthesize and release inflamentary transmitter, like IL -1β, TNF-α and IL-12, etc [4]. The activation of PRRs can also induce necroptosis and pyroptosis to stimulate inflammatory response [5]. However, an excessive immune response can aggravate corneal damage. Excessive inflammatory reaction can lead to tissue necrosis. Then necrotic cells and neutrophils infiltrating around release lysosomal enzymes, which can promote the further occurrence of necrosis and local cell lysis, leading to corneal ulcer and perforation [6].

At present, the emergence of fungal resistance makes it more difficult to cure fungal keratitis. Regrettably, there are currently no specific drugs that can not only kill drug-resistant fungi, but also reduce inflammation. Due to poor treatment, some patients will eventually become blind. Our previous experiment found that thymol could inhibit not only the growth of fungi, but also inflammation [7]. As a potential new drug in *A. fumigatus* keratitis, we further studied the anti-inflammatory mechanism of thymol.

## Materials and Methods

### Preparation of *A. fumigatus*

*A. fumigatus* strain 3.0772 was purchased from the China General Microbiological Culture Collection Center (China). First, strains were cultured on Sabouraud dextrose agar for 2-4 days. Then scrape conidia into PBS with a bacterial L-ring. Filter the suspension of pure conidia with sterile cotton gauze and adjust it to 5 × 10^7^ CFU/ml. Thymol (SelleckChem) was dissolved in DMSO, and then diluted to 1 mg/ml with DMEM-F12 medium for storage.

### Human Corneal Epithelial Cell Culture

Zhongshan Ophthalmic Center provided HCECs. Cells were cultured in DMEM at 37°C, and 5% CO_2_.

### Wound Healing Assay

HCECs (3 × 10^5^ / ml) were plated in a 6-well plate and incubated overnight at 37°C. Then scrape three parallel lines on HCECs with 200 μl sterile pipette (Corning, USA). Thymol (0 and 50 μg/ml) was pretreated in HCECs for 48 h. Before and after thymol treatment, the scratch width were surveyed by an optical microscopy (100×; Zeiss, Germany).

### In vivo Experiment

Animal models were all employed female C57BL/6 mice (8-weeks-old, Pengyue Cavens Laboratory, China). Use of mice corneas was approved by the ethics committee of the Affiliated Hospital of Qingdao University. All animal experiments adhered to the regulations of the Chinese Ministry of Science and Technology Guidelines on the Humane Treatment of Laboratory Animals (vGKFCZ-2006–398), the Declaration of Helsinki (as revised in Edinburgh 2000) and the Use of Animals in Ophthalmic and Vision Research published by the Association for Research in Vision and Ophthalmology (ARVO).

We have also established animal models to verify the safety of thymol. The mice corneas were treated with eye drops (50 μg/ml thymol or DMSO) three times one day, and then fluorescein sodium was dripped, and observed under a slit lamp through cobalt blue light (*n* = 8/group).

The mice were randomly divided into 4 groups: normal control (Group A); fungal keratitis (Group B); drug (Group C); drug-treated fungal keratitis (Group D). 8% chloral hydrate was injected into mice abdominal cavity for anesthesia. Mice right eyes were selected for subconjunctival injection with 5 μl 0.002 μg/ml DMSO (Group A and B) or 50 μg/ml thymol (Group C and D) 1 day and 2 h before infection. Conidia (2.5 μl, 5 × 10^7^ CFU/ml) was injected into the corneal stromal by a 33-gauge Hamilton syringe to establish mice fungal keratitis models. Then, mice were treated with subconjunctival injection twice a day. The corneas were observed and photographed under slit lamp (*n* = 8/group). Ocular disease was scored on the basis of Wu’s criteria [7]. Mice corneas obtained for 1 day of therapy were analyzed by Western blotting and qRT-PCR. Mice eyeballs were taken for HE staining.

### HE Staining

The eyeballs were fixed with 4% methylformaldehyde for 3 days, and then cut into 8 μm under cryostat by paraffin embedding tissue section technique. To estimate pathologic structure, the sections were stained by hematoxylin and eosin [8] and surveyed under an optical microscope.

### RNA Isolation and qRT-PCR

Total RNA was obtained by lysing corneal tissue with RNAiso Plus (Takara, Dalian, China). The RNA was quantified and reversed to obtain cDNA. Then, SYBR green, the internal reference (β-actin), and respective primers (mTLR4, mMyD88, mNF-kB, mIL-1β, mRIPK3, mMLKL, mNLRP3, mCaspase 1, mCaspase 8, mGSDMD) were added to perform the qRT-PCR. The primer pair sequences are shown in the Table 1.

### Western Blot Analysis

The Western blot protocol used was previously described [7]. The primary antibodies included anti-TLR4 (Proteintech, China), anti-MyD88 (Proteintech), anti-NF-kB (R&D Systems, U.S.A.), anti-RIPK3 (Proteintech), anti-MLKL (Proteintech), anti-NLRP3 (ZEN BIO, China), anti-Caspase1 (Novus, U.S.A.), anti-Caspase8 (Proteintech), anti-GSDMD (ABclonal, China), anti-IL-1β (R&D Systems), and anti-β-actin (Elabscience, China). An anti–rabbit (or goat or mouse or rat) antibody (ABclonal) were applied to the secondary antibody.

### Statistical Analysis

All data were analyzed with SPSS 25 software (IBM Corp.). The data are presented as the mean ± S.E.M of ≥3 independent experiments. The statistical analysis was performed using a one-way ANOVA followed by LSD-t test, which was used for analysis between two groups. *p* < 0.05 was considered to indicate a statistically significant difference. All experiments were repeated once to ensure reproducibility.

## Results and Discussion

*A. fumigatus* keratitis, as a common fungal keratitis, is a refractory keratopathy that causes the patients' visual impairment and blindness [11, 12]. Though antifungal drugs are the first-line drugs in the treatment of *A. fumigatus* keratitis, each antifungal has its advantages and boundedness, and a large proportion of patients still need active surgical intervention after the failure of drug treatment [13-15]. Our previous study had found that thymol, as a plant extract, had a curative effect on *A. fumigatus* keratitis [7]. In that article, our laboratory demonstrated that thymol not only have anti-*A. fumigatus* activity, but also inhibited the LOX-1/ IL-1β signal. This experiment further demonstrated thymol’s role of inhibiting inflammatory and inflammatory death in *A. fumigatus* keratitis.

**Fig. 1:**
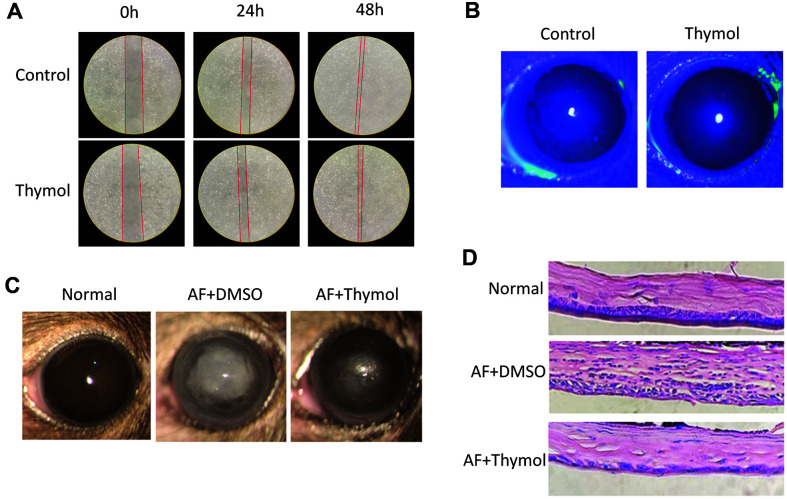
Thymol relieved inflammation of *A. fumigatus* keratitis. (**A**) Wound healing assay of thymol on reepithelization potentiality. (**B**) Photographs of mice corneas fluorescein sodium staining were taken by a slit lamp. (**C**) Representative photographs were taken and clinical scores were evaluated under a slit lamp at one day post-infection. (**D**) HE staining of corneal tissue sections (magnification 400x) of DMSO or thymol treated *A. fumigatus* keratitis mice.

We verified 50 μg/ml thymol’s safety again. Wound healing assay revealed that 50 μg/ml thymol had no significant effect on cells migration within 48 h (Fig. 1A). In other words, it was shown that 50 μg/ml thymol treatment did not affect cell migration from the limbus to the ulcer site, and this concentration did not affect cell proliferation, which further verifies the credibility of our previous experiments [7].

Neither the control group nor the thymol eye drop group caused yellow-green fluorescein sodium staining within 5 days, indicating that 50 μg/ml thymol would not cause corneal epithelial damage in mice (Fig. 1B). This added that neither thymol eye drops nor subconjunctival injections [7] caused loss of corneal tissue. Thus, all subsequent experiments followed this concentration.

We previously found that the corneas were more transparent and less inflammatory in the thymol-treated group in contrast to natamycin-treated group [7]. In order to survey the potency of thymol, we recorded and take pictures using slit lamp (Fig. 1C). Obviously, keratitis was significantly alleviated after thymol therapy. By contrast with DMSO-injection group, the thymol-injection group had smaller ulcer area, lower degree of edema, and higher transparency. This was consistent with the clinical score. The clinical scores in thymol-injection group was obviously lower. This provides a possibility for thymol to replace antifungal drugs.

Fungus invasion causes innate and adaptive immune-mediated inflammation, leading to subsequent cornea tissue necrosis in the surrounding area [4, 11, 16]. Toll-like receptor 4 (TLR4) recognizes cell wall components of *A. fumigatus* [17], and recruits myeloid differentiation factor 88 (MyD88) molecules, which then causes nuclear factor-kappa B (NF-kB) to translocate to the nucleus and express pro-inflammatory and chemotactic cytokines [16,18,19]. These include CXCL8, CCL2, CCL3, and *et al* [20, 21], which accumulates macrophages and neutrophils from limbus to infection site, and pro-IL-1β, which need to be actived [22-24].

Although inflammatory cells were essential for killing fungi, excessive could also cause tissue damage and lose corneal transparency [25-27]. In previous experiment, we performed immunofluorescence staining on neutrophils and macrophages and found that thymol significantly reduced their recruitment in keratitis [7]. In this experiment, H&E staining showed that corneal sections in thymol-treated group significantly had less inflammatory cells infiltration (Fig. 1D).

Although we previously demonstrated that thymol exerted anti-inflammatory results by suppressing the LOX-1/ IL-1β signal, the mechanism of thymol controlling other inflammatory pathways in *A. fumigatus* keratitis was still unclear. The regulation and release of IL-1β is divided into two processes. The first is to initiate initial activation by PRRs such as TLR4 to produce inactive 31 kD pro-IL-1β [28, 29]. The second observation promotes the lysis of pro-IL-1β into biologically active IL-1β through NLRP3/ ASC/ caspase-1 inflammasome [30, 31]. Then Caspase-1 and caspase-8 lyse Gasdermin D (GSDMD) to cause pyroptosis, IL-1β is secreted from the pores of pyroptosis cells to the infection site [32, 33].

**Fig. 2:**
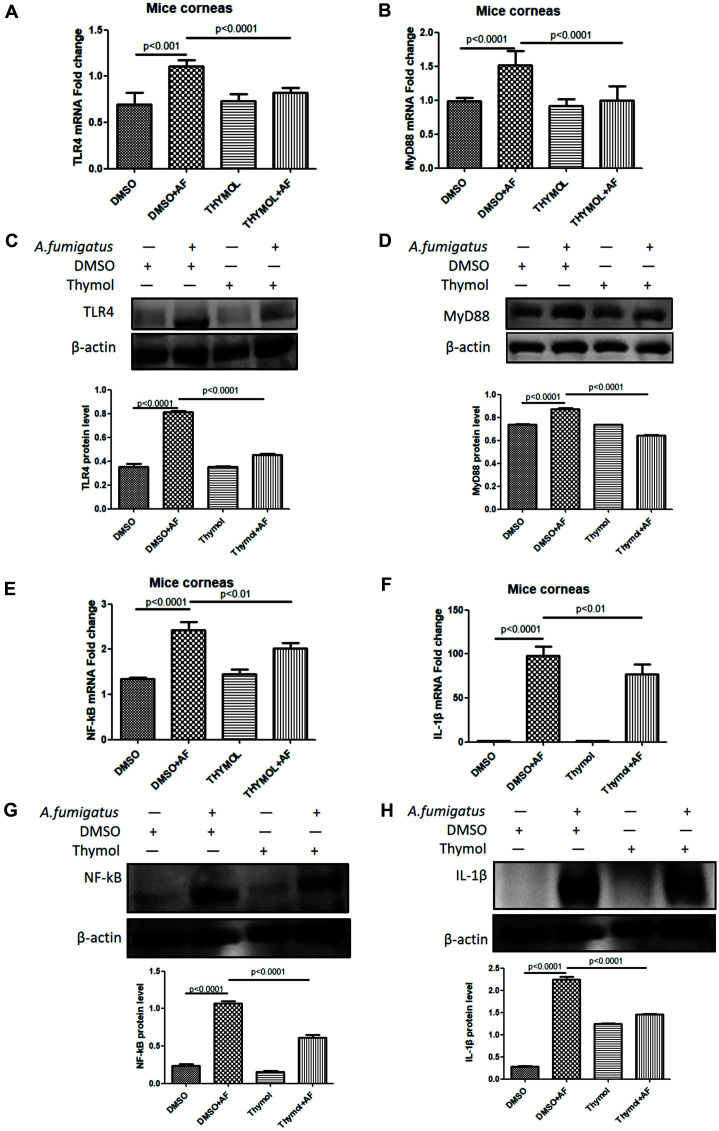
Thymol downregulated the TLR4/ MyD88/ NF-kB/ IL-1β signal expression in *A. fumigatus* keratitis. After treatment with thymol, the mRNA levels of TLR4 (**A**), MyD88 (**B**), NF-kB (**E**) and 17KD IL-1β (**F**) in the cornea were decreased. The thymol group had lower TLR4 (**C**), MyD88 (**D**), NF-kB (**G**) and IL-1β (**H**) protein concentrations compared to the DMSO group.

To further research thymol’s anti-inflammatory functions in *A. fumigatus* keratitis, qRT-PCR (Figs. 2A, 2B, 2E, and 2F) and Western blotting (Figs. 2C, 2D, 2G, and 2H) were used to detect inflammatory signal in keratopathy. The results showed the production of TLR4, MyD88, NF-kB and IL-1β was significantly down-regulated in both mRNA and protein levels after 1 day treatment of *A. fumigatus* keratitis with thymol. This seemed to suggest that thymol reduced the expression of IL-1β by downregulating the TLR4/ MyD88/ NF-kB/ IL-1β signal, which further exerted anti-inflammatory effect.

Since the viability of *A. fumigatus* was significantly decreased at the tested concentration [7], it is not certain that reduced TLR4/MyD88/NF-kb was originated from thymol treatment per se or the decreased viability of *A. fumigatus*. In order to prove thymol’s anti-inflammatory property, we supplemented in vitro experiments. HCECs were pretreated with thymol for 24 h, while DMSO pretreating in control group. Before *A. fumigatus* conidia stimulating HCECs, remove the original medium, wash 3 times with PBS, and then re-add the medium without thymol. Then add conidia (5 × 106 CFU/ml) to stimulate HCECs 8 or 16 h. Then collect cells for qRT-PCR. After pretreating HCECs with thymol, the mRNA of TLR4/MyD88/NF-kB were also down-regulated (Fig. S1). However, in previous study [7], we have found that thymol could not only kill fungi, but also exert anti-inflammatory property in the treatment of *A. fumigatus* keratopathy. In this article, we mainly want to explore the anti-inflammatory property of thymol in fungal keratopathy.

**Fig. 3:**
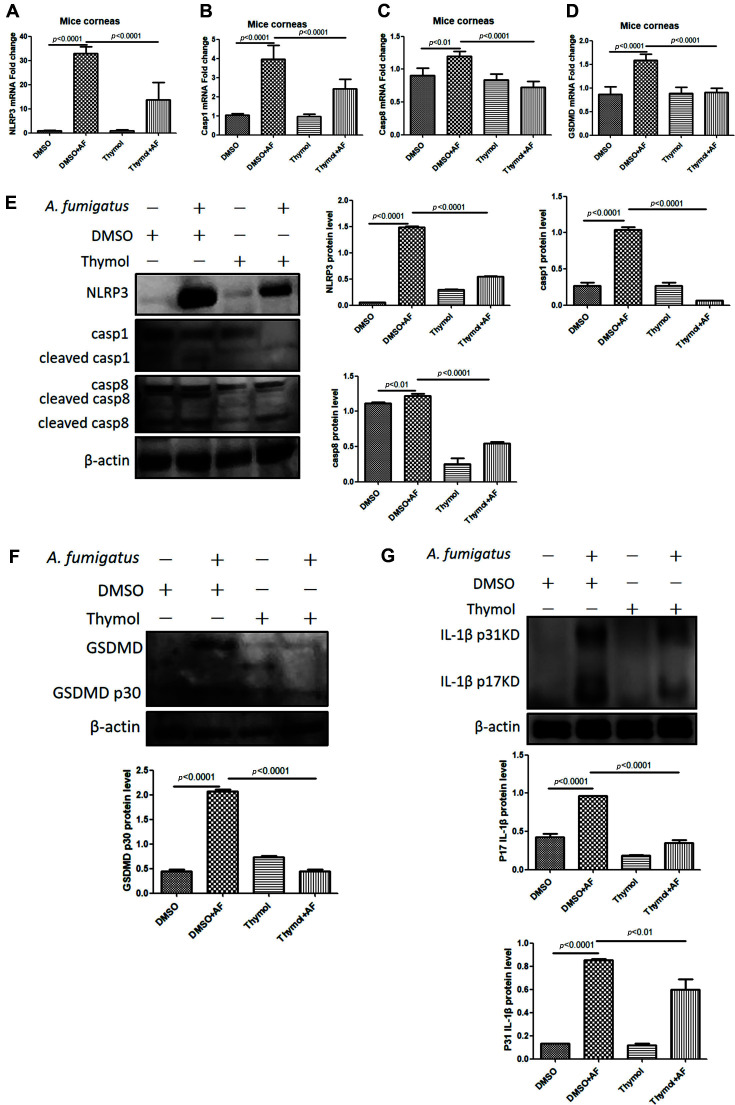
Thymol suppressed pyroptosis. After thymol treatment, the mRNA (**A, B, C, D**) and protein (**E, F, G**) levels of NLRP3, cleaved-Caspase 1, cleaved-Caspase 8, N-GSDMD and 17KD IL-1β in mice with fungal keratitis were significantly decreased.

Pyroptosis is another programmed cell death mediated by gasdermin, which its markers are inflammatory caspase and pro-inflammatory mediators, thus activating a strong inflammatory response [34, 35]. The type of cell death induced by caspase is affected by substrate specificity and downstream signaling molecules [35]. Different from apoptosis, pyroptosis is an inflammatory death. Intracellular pro-inflammatory factors including IL-1 family can be released through pyroptosis cells. Previous studies have shown that Caspase1 inhibitor therapy could reduce *Pseudomonas aeruginosa* keratitis [36]. Another found caspase8 activated NLRP3 inflammasome and increased IL-1β’s generation in acute glaucoma [37]. Our laboratory also found that GSDMD participated in the prophase immune process of *A. fumigatus* keratitis. In addition, inhibition of GSDMD could reduce inflammation by blocking caspase1 signaling pathway to affect IL-1β secretion and neutrophil and macrophage accumulation [37]. We also detected the pyroptosis-related mRNA (Figs. 3A-3D) and protein (Figs. 3E-3G) levels. Compaired with DMSO-treated subset, the secretion of NLRP3, cleaved-Caspase1, cleaved-Caspase8, N-GSDMD and 17KD IL-1β were obviously decreased in thymol-treated subset. Our results showed that Thymol suppressed pyroptosis.

**Fig. 4:**
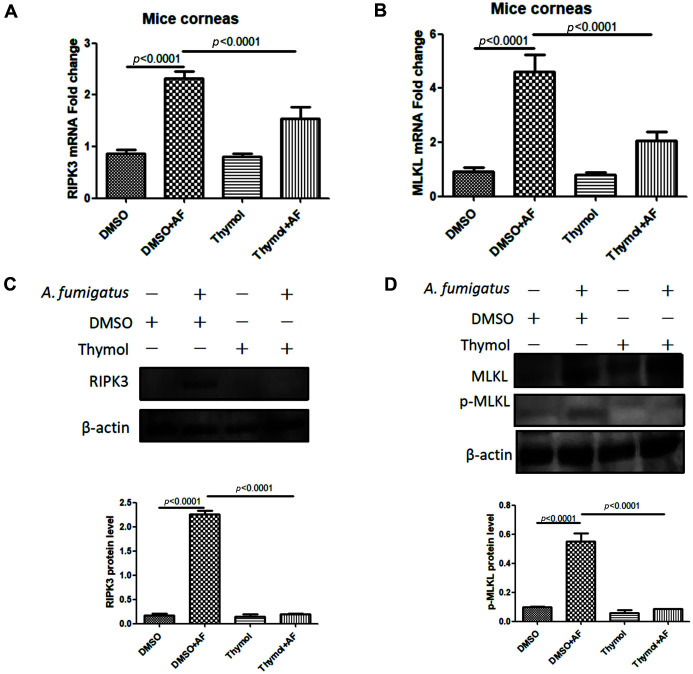
Thymol suppressed necroptosis. After thymol treatment, the mRNA (**A, B**) and protein (**C, D**) levels of RIPK3 and p-MLKL in mice with fungal keratitis were significantly decreased.

Necroptosis which is another death depends on RIPK1 and RIPK3 kinases [38]. Death signal induces activation of necroptosis-specific executive proteins RIPK3 and p-MLKL [39]. P-MLKL is oligomerized and translocated to cell membrane structure, resulting in cell death [40]. The death of infected cells is accompanied by the secretion and release of cell contents, which will further induce local immune inflammatory response. While recruiting inflammatory cells, it participates in activating and regulating the adaptive immune response [41]. It was found that p-MLKL inducesd the formation of NLRP3 inflammasome by inducing K[+] efflux, thus promoting the maturation of IL-1β [42]. Our experiment found that in thymol-treated subset, corneas expressed lower levels of RIPK3 and p-MLKL than that in DMSO-treated subset (Figs. 4A-4D). Therefore, it was speculated that thymol treatment reduced the programmed cell necroptosis of cornea and local immune inflammatory response in mice, which made the cornea more transparent.

In summary, we revealed thymol’s anti-inflammatory mechanism on *A. fumigatus* keratitis. Thymol had therapeutic effect on *A. fumigatus* keratitis by controlling inflammatory cells recruitment, and the TLR4/ MyD88/NF-kB/ IL-1β signal expression. In addition, thymol could reduce inflammatory death to inhibit of IL-1β activation and release, whether it is NLRP3/ caspase1/ caspase8/ GSDMD pathway mediated pyroptosis or RIPK3/ MLKL pathway mediated necroptosis. These findings supported thymol as an innovative drug for therapy of fungal keratitis.

## Supplemental Materials

Supplementary data for this paper are available on-line only at http://jmb.or.kr.

## Figures and Tables

**Table 1:** Nucleotide sequences of primers for real-time RT-PCR.

Gene	Primer sequence (5’–3’)
mβ-actin	F: GAT TAC TGC TCT GGC TCC TAG C R: GAC TCA TCG TAC TCC TGC TTG C
mIL-1β	F: CGC AGC AGC ACA TCA ACA AGA GC R: TGT CCT CAT CCT GGA AGG TCC ACG
mRIPK3	F: CTT CAG AGG CAC AAC ACC TGG R: CCC TGT CAT TGG ATT CGG TGG
mMLKL	F: AGG ATT GCC CTG AGT TGT TGC R: TGT CCG TGG ATT CTT CAA CCG
mNLRP3	F: TGC CTG TTC TTC CAG ACT GGT GA R: CAC AGC ACC CTC ATG CCC GG
mCaspase1	F: GCC TGG TCT TGT GAC TTG GA R: ATG TCC CGG GAA GAG GTA GA
mCaspase8	F: ACA AAC CTC GGG GAT ACT GTC R: AGT GCA GTC GTC GTA AGA TAC TA
mGSDMD	F: TCG CTT GGT GGA CCC AGA TAC R: AGG CTG TCC ACC GGA ATG AG
mTLR4	F: CGC TTT CAC CTC TGC CTT CAC TAC AG R: ACA CTA CCA CAA TAA CCT TCC GGC TC
mMyD88	F: AGC AGA ACC AGG AGT CCG AGA AGC R: GGG GCA GTA GCA GAT AAA GGC ATC G
mNF-kB	F: GCT TTG CAA ACC TGG GAA TA R: TCC GCC TTC TGC TTG TAG AT
