# Supplementary material for: Thymol Ameliorates Aspergillus fumigatus Keratitis by Downregulating the TLR4/ MyD88/ NF-kB/ IL-1β Signal Expression and Reducing Necroptosis and Pyroptosis
Source: J Microbiol Biotechnol. 2022 Dec 1;33(1):43–50. doi: 10.4014/jmb.2207.07017 (PMC9895997; doi:10.4014/jmb.2207.07017)
Supplement: Supplementary file 1 [file jmb-33-1-43-supple.pdf]

J. Microbiol. Biotechnol.
https://doi.org/10.4014/jmb.2207.07017

Supplementary fig. S1

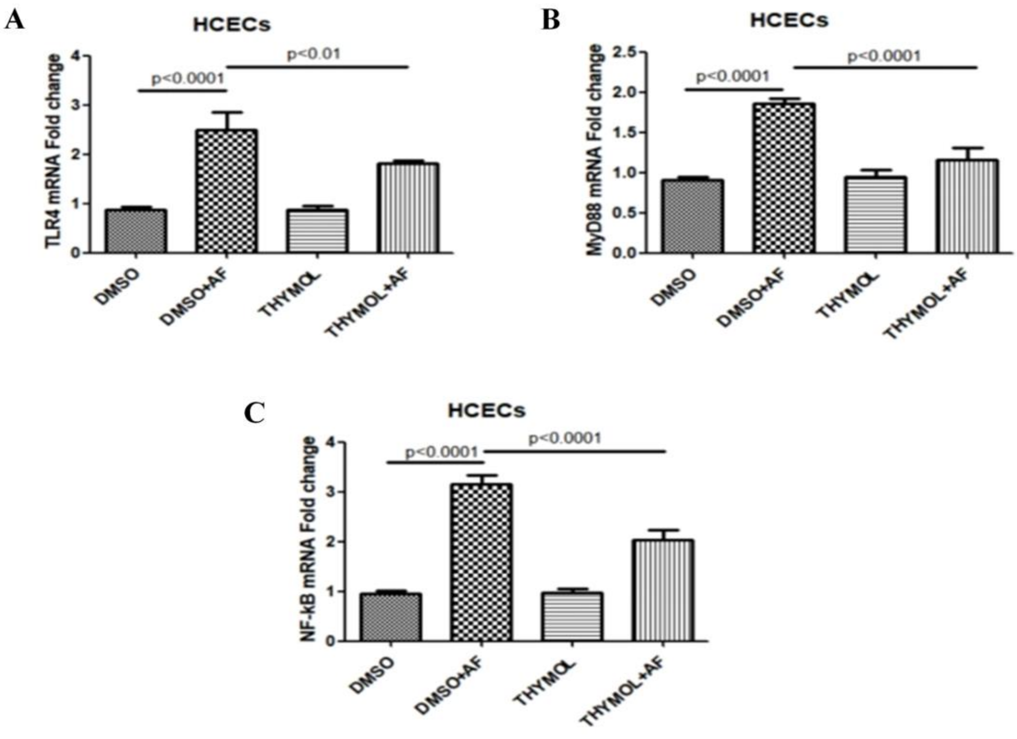

Supplementary Fig. S1 After thymol pre-treatment, the mRNA (**A, B, C**) of TLR4, MyD88, NF-kB in HCECs stimulated with *A. fumigatus* conidia were significantly decreased.
